# Mucocutaneous Manifestations Among HIV-Infected Patients in Madagascar: Cross-Sectional Study

**DOI:** 10.2196/47199

**Published:** 2023-08-15

**Authors:** Fandresena Arilala Sendrasoa, Volatiana Mercia Falimiarintsoa, Lala Soavina Ramarozatovo, Fahafahantsoa Rapelanoro Rabenja

**Affiliations:** 1 Département de Dermatologie Faculté de Médecine Université d'Antananarivo Antananarivo Madagascar

**Keywords:** HIV infection, mucocutaneous manifestations, oral candidiasis, HIV, cross-sectional study, lesion, mucocutaneous, dermatitis, sarcoma, syphilis, immune, useful, disease

## Abstract

**Background:**

More than 90% of HIV-infected patients present with at least one mucocutaneous manifestation during the course of their disease. Insufficient data are available regarding dermatologic findings among HIV-infected patients in Madagascar.

**Objective:**

This study aimed at evaluating the spectrum of mucocutaneous manifestations and their relationship with CD4 cell counts in HIV-infected patients in Madagascar.

**Methods:**

A cross-sectional study on HIV-positive patients attending the Department of Infectious Diseases in the University Hospital of Antananarivo in Madagascar was conducted from January 2013 to March 2020. HIV-positive patients older than 18 years and receiving antiretroviral therapy as well as those awaiting antiretroviral therapy commencement were included.

**Results:**

Among 328 patients enrolled in this study, 167 (51%) presented with at least one type of mucocutaneous lesion. Oral candidiasis was the most common presentation, followed by seborrheic dermatitis and Kaposi sarcoma. Decreases in CD4 cell counts were substantially correlated with oral candidiasis, syphilis, and condyloma acuminatum.

**Conclusions:**

According to our findings, oral candidiasis, syphilis, and condyloma acuminatum may serve as clinical indicators for predicting the immune status of patients. As HIV infection progressed and immune function declined, an increase in cutaneous manifestations was observed.

## Introduction

In Madagascar, the prevalence of HIV infection in the general population increased from 0.02% to 0.25% between 1989 and 2018. High prevalence of HIV was found among key populations in Madagascar, with rates of 14.8% in men who have sex with men, 8.4% in injecting drug users, and 5.6% in sex workers [1,2]. In 2019, an estimated 13% of people living with HIV (PLWH) in Madagascar had access to antiretroviral therapy (ART). Although the number of people with access to ART had increased, 1400 individuals died from AIDS-related diseases [3].

HIV infection is commonly associated with various mucocutaneous manifestations that can be present in all situations. Mucocutaneous disorders develop in more than 90% of HIV-infected individuals at some point during the course of their illness [4]. The epidemiologic profile of dermatologic illnesses related to HIV varies among countries. Prevalence reported by Asian [5,6] and African studies [7,8] ranged from 52.5% to 96%. The spectrum of dermatologic illnesses related to HIV has also changed since the introduction of highly active ART [9].

The purpose of this study was to describe the epidemiology and the clinical spectrum of mucocutaneous manifestations in HIV-positive patients attending the Department of Infectious Diseases in the University Hospital of Antananarivo in Madagascar.

## Methods

### Ethics Approval

This study was approved by the Ethics Commission of the University Hospital of Antananarivo in Madagascar (23-CHUJRB/CE). Confidentiality was maintained. Patients provided written informed consent to allow their medical records data to be used in research and their case details to be published.

### Procedure

A cross-sectional study was conducted in HIV-positive patients (men and women aged ≥18 years) seeking care at the Department of Infectious Diseases at the University Hospital of Antananarivo in Madagascar from January 2013 to March 2020. Patients receiving ART and those awaiting ART commencement were included.

Data on the mode of infection, duration of disease, the nearest level of CD4 cell counts (within the last 2 months) at the time of diagnosis of a specific skin condition, treatment regimen, and any prior dermatological history were considered. Physical examination of patients was conducted by dermatologists. Dermatoses were primarily diagnosed clinically, with additional support from mycological, histological, and hematological testing, as necessary.

### Statistical Analysis

Statistical analysis was performed with Epi Info software (version 3.5.4; Centers for Disease Control and Prevention). Categorical data were compared using the chi-square test, and the student *t* test (2-tailed) was used to analyze continuous variables. The level of statistical significance was set at *P*<.05. Regression analysis was performed to assess the relationship between the number of dermatoses and CD4 cell counts.

## Results

Among the 345 HIV-positive patients seen during the study period, 7 were excluded due to incomplete data, and an additional 10 declined participation. As a result, a total of 328 patients were included in the study, of whom 215 (65.5%) of them were male. A total of 168 (51%) patients presented with at least one dermatological disease. Among these 168 patients, 130 (77.3%) were receiving ART. Of the total 328 participants, 123 (37%) patients in the age group of 30-39 years were affected by HIV. The median age was 41 (IQR 27-52) years. Among the 328 participants, the primary mode of HIV transmission was heterosexual in 272 (83%) individuals, whereas the number of men who have sex with men was 30 (9.1%). In terms of occupation, 104 (32%) patients worked in the commerce sector, 37 (11.2%) were housewives, 26 (8%) were students, and 8 (2.4%) were health care professionals. The average duration of HIV infection was 9.8 (SD 6.2) years. Additionally, 160 (48.7%) patients had a low level of education.

**Figure 1:**
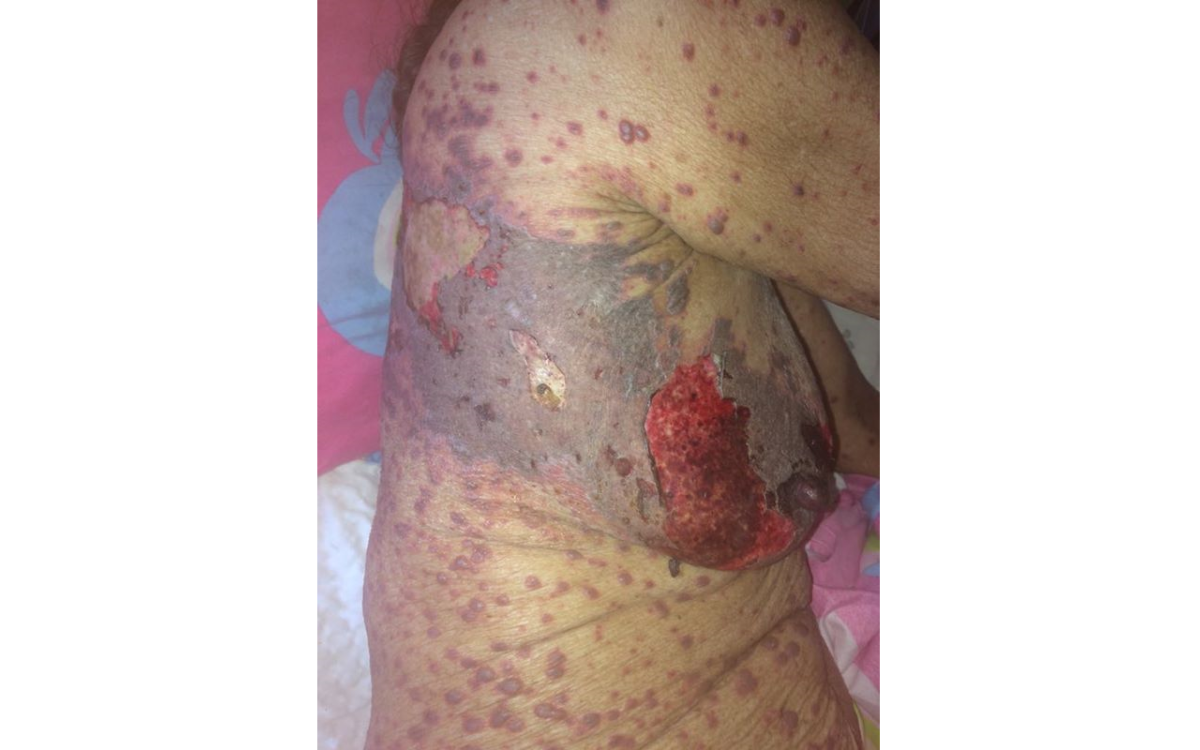
Hemorrhagic and extensive herpes zoster.

**Figure 2:**
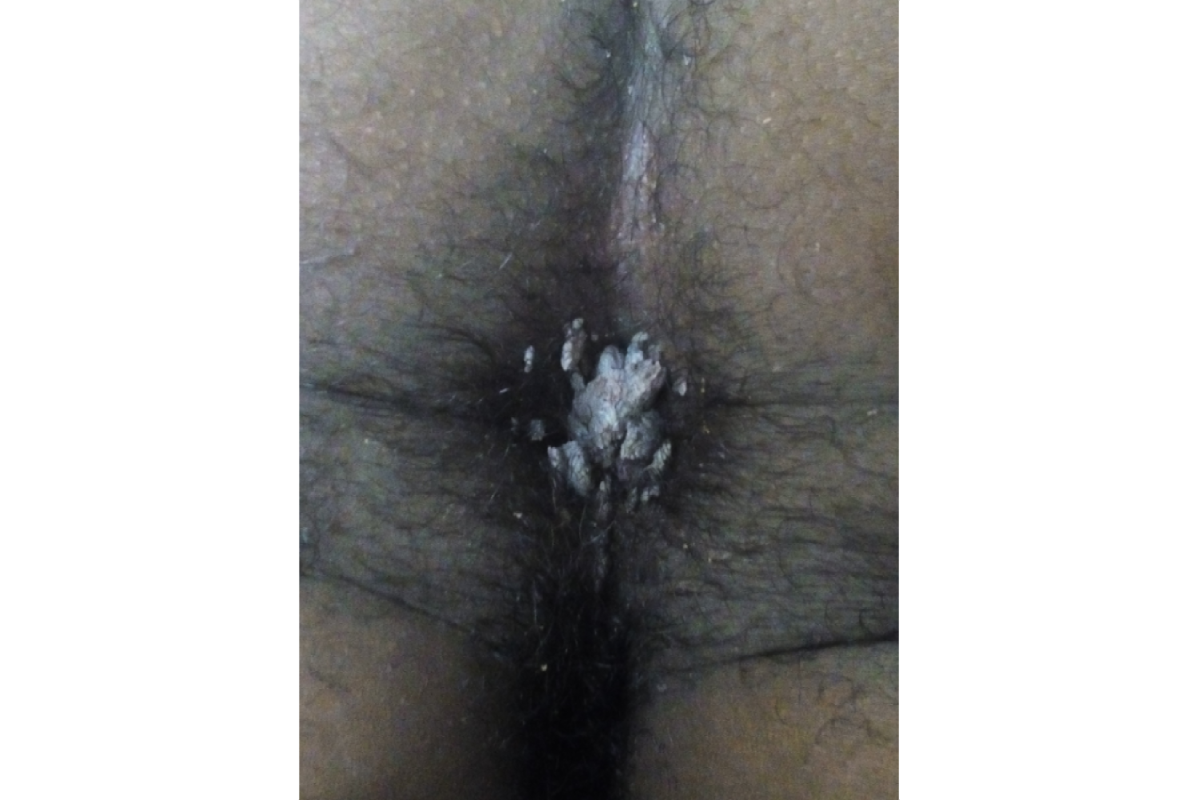
Anal condylum acuminatum.

A total of 231 dermatoses were identified in 168 HIV-infected patients. Among these patients, 117 (69.6%) had one type of dermatoses, while 41 (24.4%), 8 (4.7%), and 2 (1.1%) presented with 2, 3, and 4 types of dermatoses, respectively. Infectious dermatoses affected half of the participants, with 111 (66%) patients presenting with fungal infections, which were the most prevalent, followed by viral infections in 35 (20.8%) patients and bacterial infections in 17 (10%) patients. The most common fungal infection was oral candidiasis, while herpes zoster (Figure 1) and syphilis were the most common viral and bacterial infections, respectively. Additionally, 10 (5.9%) of 168 patients presented with condyloma acuminatum (Figure 2).

**Figure 3:**
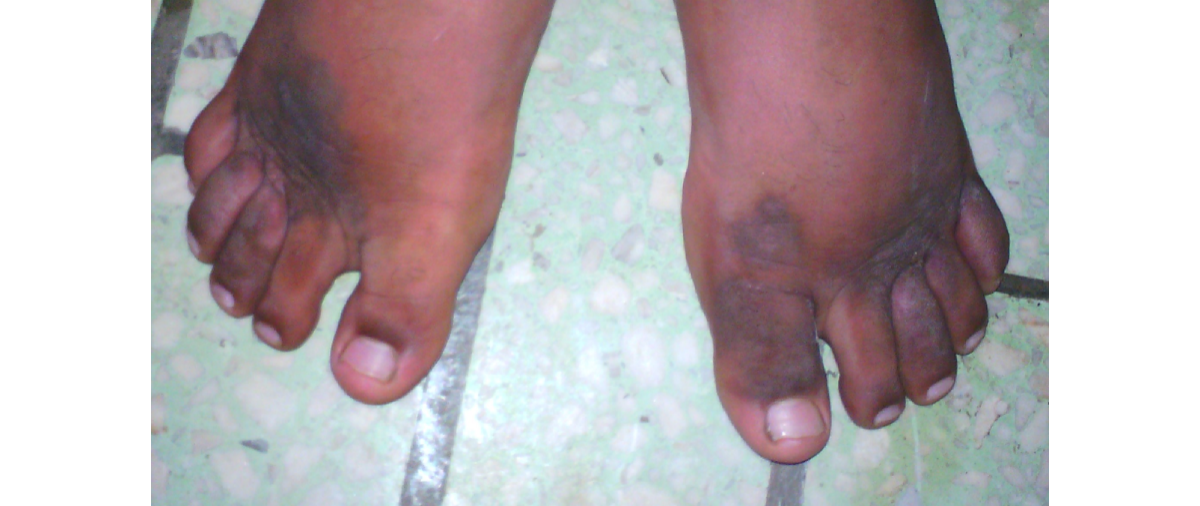
Kaposi sarcoma affecting lower limbs.

**Figure 4:**
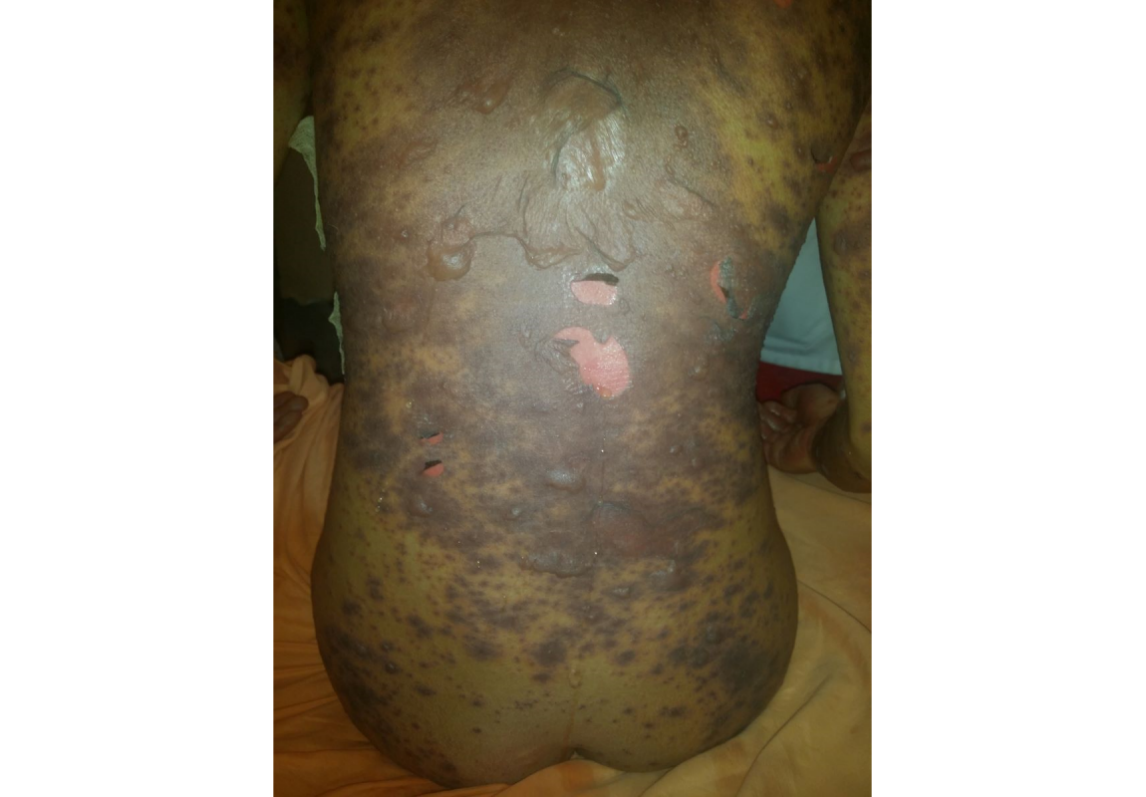
Lyell syndrome induced by antiretroviral therapy.

Noninfectious dermatoses affected 61 (36.3%) of the 168 patients. Among the noninfectious dermatoses, inflammatory dermatoses was the most prevalent, with seborrheic dermatitis being the main condition observed in 27 (11.6%) patients. Regarding neoplastic diseases, 16 patients presented with Kaposi sarcoma (8 men and 8 women), with 6 patients in the age group of 30-39 years (Figure 3). No case of other types of skin tumors was seen. Toxidermia (Figure 4) was observed in 7 patients, 2 of whom were induced by ART and the other 5 were attributed to antituberculosis drugs and trimethoprim-sulfamethoxazole. Exanthematous rash, the most common type of drug eruption, was seen in 6 cases. Additionally, 1 case of acute generalized exanthematous pustulosis was observed.

Table 1 shows a list of the most common dermatologic diagnoses encountered in this study, with the corresponding mean CD4 cell counts.

The mean CD4 cell count for all patients was 190 (SD 32) cells/mm^3^. LTCD4 was less than 200 cells/mm^3^ in 139 (83%) of 168 patients who had skin manifestations. The mean CD4 cell count of patients with cutaneous manifestations (119, SD 40 cells/ mm^3^) was significantly lower than the mean CD4 cell count of those without manifestations (262, SD 65 cells/mm^3^; *P*<.05).

Of the 328 PLWH in this study, 259 (79%) were receiving ART. Among them, 251 (77%) were receiving a fixed-dose combination of 2 nucleoside reverse transcriptase inhibitors and nonnucleoside reverse transcriptase inhibitors. However, 47 (18%) of the 259 patients had not initiated treatment. Of the 8 PLWH, 2 (3%) who were treated by 2 nucleoside reverse transcriptase inhibitors associated with protease inhibitors were pregnant.

Among the infectious dermatoses, oral candidiasis (*P*=.001), syphilis (*P*=.002), and condyloma acuminatum (*P*=.03) showed statistically significant associations with the CD4 count (<200 cells/mm^3^). The association of dermatoses with CD4 cell counts is shown in Table 2.

**Figure 5:**
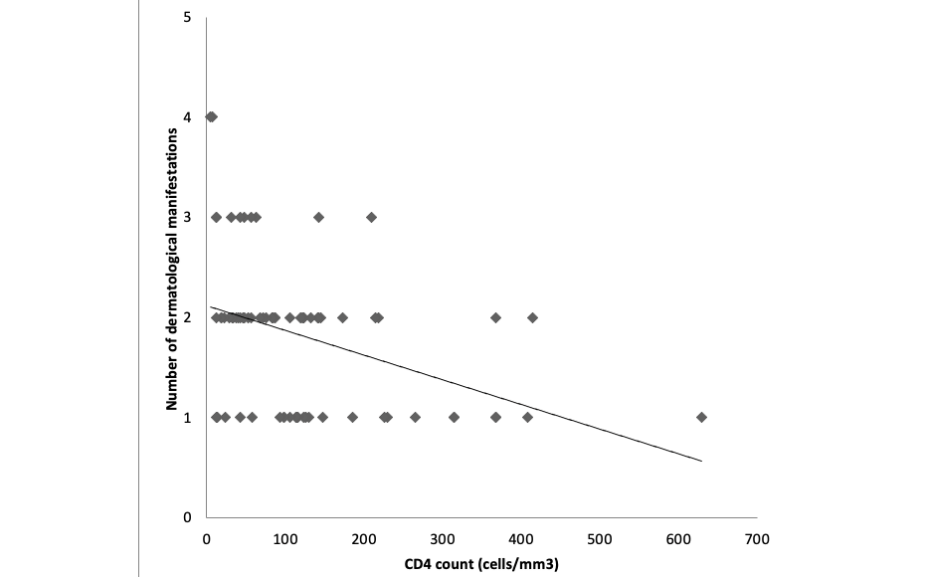
The number of dermatoses according to CD4 cell counts. CD4 cell counts were inversely proportional to the number of dermatoses presented by people living with HIV, with a strong negative correlation between these two parameters (correlation coefficient –0.39).

CD4 cell counts were inversely proportional to the number of dermatoses presented by PLWH—as the CD4 cell counts increased, the number of dermatoses decreased (Figure 5). A strong negative correlation was identified, with a correlation coefficient of –0.39.

**Table 1:** The most common dermatologic diagnoses among HIV-positive patients, along with the corresponding mean CD4 cell counts.

Dermatologic diagnoses			Participants (n=231), n (%)		CD4 count (cells/mm^3^), mean (SD)
**Infectious diseases**					
	Oral candidiasis	111 (48.2)		97 (80)	
	Herpes zoster	12 (5.2)		164 (70)	
	Syphilis	11 (4.7)		187 (98)	
	Genital herpes	10 (4.3)		105 (79)	
	Condyloma acuminatum	10 (4.3)		217 (101)	
	Molluscum contagiusum	2 (0.86)		78 (31)	
	Pustulosis	2 (0.86)		226 (57)	
	Furunculosis	3 (1.30)		186 (61)	
**Noninfectious diseases**					
	Kaposi sarcoma	16 (6.9)		68 (43)	
	Seborrheic dermatitis	27 (11.6)		88 (49)	
	Prurigo	13 (5.6)		106 (71)	
	Toxidermia	7 (3)		163 (78)	

**Table 2:** Association of dermatoses with CD4 cell counts.

Dermatologic diagnoses	CD4<200 cells/mm^3^ (n=186), n (%)	CD4≥200 cells/mm^3^ (n=24), n (%)	*P* value
Oral candidiasis	103 (55.3)	8 (33.3)	.001
Herpes zoster	9 (4.8)	3 (12.5)	.33
Genital herpes	8 (4.3)	2 (8.3)	.66
Syphilis	6 (3.2)	5 (20.8)	.002
Condyloma acuminatum	6 (3.2)	4 (16.6)	.03
Kaposi sarcoma	16 (8.6)	0 (0)	.29
Seborrheic dermatitis	26 (13.9)	1 (4.1)	.23
Prurigo	12 (6.4)	1 (4.1)	.57

## Discussion

### Principal Findings

The prevalence of mucocutaneous manifestations in patients with HIV was 51% in our study. The mean CD4 cell count (119 cells/µl) of patients with cutaneous manifestations was significantly lower than the mean CD4 cell count of those without manifestations. Oral candidiasis was the most prevalent of infectious dermatoses and was associated with a mean CD4 count of 97 cells/mm^3^. Seborrheic dermatitis was the most prevalent of noninfectious dermatoses. The prevalence of mucocutaneous manifestations in HIV-infected patients found in this study is close to that among Korean patients (55.6%), reported by Kim et al [5]. This observed similarity may be contributed to changes in the prevalence of cutaneous disorders associated with the introduction of ART. Previous studies have found dermatological manifestations to be less prevalent in patients treated by ART compared with those who did not receive ART [10,11]. However, comparing the prevalence of mucocutaneous manifestations in HIV-infected patients is challenging due to differences in study designs and the socioeconomic status of patients included in different studies.

The mean age of our study participants was 36.5 (SD 19.1) years. This finding is consistent with results reported by Josephine et al [12] for patients in Cameroon (mean age 37.2 years) and Atadokpede et al [13] for patients in Benin (mean age 37.8 years). According to data from the 4th Demographic and Health Survey in Madagascar conducted in 2008-2009 [14], some factors may contribute to the early age of HIV infection among the participants in this study, including low levels of education, lack of access to health care services and AIDS education, as well as low condom use, particularly among young people aged 15-19 years.

Patients with cutaneous manifestations had a significantly lower mean CD4 cell count (119 cells/µl) compared to patients without signs of cutaneous manifestations (262 cells/µl; *P*<.05). Our results are consistent with those reported by Lahoti et al [15] from India and Li et al [6] from China.

Among the infectious dermatoses, oral candidiasis (*P*=.001), syphilis (*P*=.002), and condyloma acuminatum (*P*=.03) showed statistically significant correlations with CD4 cell counts. Several authors reported the association of oral candidiasis with low CD4 cell counts [1,16,17]. In addition, Veldhuijzen et al [18] have demonstrated a relationship between low CD4 cell counts, increased HPV infection rates, and the development of anogenital warts. However, Azfar et al [19] were unable to show a relationship between skin manifestations and CD4 cell counts. These discrepancies in the results may be attributed to variations in sample sizes, disease stages, infection routes, and regional patterns of the reported.

A negative correlation was found between CD4 cell counts and the number of dermatoses seen in PLWH. This finding suggests that the number of mucocutaneous diseases related to HIV infection should be considered among the key clinical indicators for the prediction of underlying immune status and disease progression. Asian and Indian studies have reported similar results [20,21].

Oral candidiasis was found to be the most prevalent infectious dermatoses, associated with a mean CD4 count of 97 cells/mm^3^. Our result is consistent with results reported in previous studies [8,22], which have also reported a high prevalence of oral candidiasis in PLWH, ranging from 36% to 88%. A statistically significant correlation was found between a CD4 count <200 cells/mm^3^ and the frequency of oral candidiasis (*P*<.001). Our results are consistent with those reported by Monsel et al [8] from Senegal, Ghate et al [23] from India, and Suryana et al [24] from Indonesia. This indicates that the presence of fungal infections is a predictor of advanced immunosuppression in HIV infection.

Among 231 dermatological diagnoses, seborrheic dermatitis was found to be the most prevalent of noninfectious dermatoses, presented by 27 (11.73%) patients. Our result is consistent with the findings reported by Claasens et al [25] in South Africa. A high frequency of seborrheic dermatitis (40%-80%) associated with HIV infection was reported before the use of highly active ART [26]. However, studies conducted in the ART era in China, India, and the United States have reported low prevalence rates of seborrheic dermatitis [9,27,28].

Human herpes virus type 8 (HHV-8) is the causative agent of Kaposi sarcoma. The seroprevalence of HHV8 in Africa ranged from 2% to 100% depending on the regions [29]. Kaposi sarcoma was presented by 16 (6.95%) patients in our study, with a mean CD4 count of 68 cells/mm^3^. Ranaivo et al [30] reported lymphedema of the upper limb as a revealing symptom of Kaposi sarcoma associated with HIV infection. Environmental factors may also influence the onset of this disease. Similar to what is observed in other African countries, our study confirms the occurrence of Kaposi sarcoma in the advanced stages of immunosuppression. However, Kaposi sarcoma is relatively rare among the US population and Asians due to the lower seroprevalence of HHV8 in these populations [9,31].

Another important skin problem observed in our study was prurigo, which was present in 13 (5.65%) cases and associated with a mean CD4 count of 106 cells/mm^3^. In African populations, prurigo has been linked to arthropod bites and poor socioeconomic conditions [13,32]. Our study showed that prurigo was predictive of advanced immunosuppression.

Herpes zoster was seen in 12 (5.21%) patients, with a mean CD4 count of 164 cells/mm^3^. ART has been shown to reduce the incidence of herpes zoster in adults with HIV, presumably because of immune restoration [33]. Herpes zoster can occur in adults with HIV at any CD4 lymphocyte cell count, but the risk of disease is higher with CD4 counts <200 cells/mm^3^. Several studies have also reported that the risk of herpes zoster is increased in the 6-month period immediately after initiation of ART [34,35].

Toxidermia was observed in 7 (3.10%) patients among our study population, with benign drug eruptions accounting for 6 of these toxidermia cases. Our result is close to those reported in South Africa by Claasens [25]. However, Asian studies in the ART era have reported higher prevalence rates for drug eruptions, ranging from 10% to 17% [6,9,20]. The mean CD4 count of these patients was 163 cells/mm^3^. This finding is consistent with the results reported by Goh et al [20] in Singapore and Hagos et al [36] in Eritrea, which demonstrated that a low CD4 cell count was associated with adverse drug eruptions.

### Conclusions

Our study provides data on the epidemiology and spectrum of mucocutaneous diseases in patients with HIV infection attending the Department of Infectious Diseases at the University Hospital of Antananarivo in Madagascar. Oral candidiasis, seborrheic dermatitis, and Kaposi sarcoma were found to be the most prevalent dermatoses. Among 328 participants included, toxidermia was observed in 7 (3.1%) patients. Oral candidiasis, syphilis, and warts showed potential as useful clinical indicators for predicting the immune status of the patients.

## Abbreviations

ART: antiretroviral therapy
HHV-8: human herpes virus type 8
PLWH: people living with HIV
